# The So-Called Pre-Clinical Rheumatoid Arthritis: Doubts, Challenges, and Opportunities

**DOI:** 10.3390/jcm13216387

**Published:** 2024-10-25

**Authors:** Raimon Sanmartí, Beatriz Frade-Sosa, Andres Ponce

**Affiliations:** Arthritis Unit, Rheumatology Department, Hospital Clinic of Barcelona, 08036 Barcelona, Catalonia, Spain; frade@clinic.cat (B.F.-S.); aponce@clinic.cat (A.P.)

A Clinical Case of Possible Pre-Rheumatoid Arthritis

A 46-year-old female, a scientist working in a laboratory, presented in the Arthritis Unit with polyarthralgia of an intermittent course. She has a cousin with rheumatoid arthritis (RA) but denied other inflammatory rheumatic diseases in her family. She has a history of urticaria “a frigore”. The current symptoms began one year ago with mild bilateral metatarsalgia, with a relapsing course. Three months before the first visit, she complained of bilateral shoulder pain, and three weeks later, polyarthralgia, particularly in her hands, without swelling, which improved after 2 weeks and was treated with ibuprofen. The joint pain disappeared but recurred after one month, affecting both hands, especially in the proximal interphalangeal (PIP) and metacarpophalangeal (MCP) joints. The symptoms predominated in the early morning, with mild morning stiffness (<60 min). Her family physician prescribed ibuprofen with partial improvement. Initial blood tests showed normal ESR and CRP values, negative rheumatoid factor (RF), and antinuclear antibodies (ANA) but high levels of anti-CCP antibodies (CCP2 test: 112 IU, normal value <3). The clinical exam at the first visit showed a positive squeeze test but no joint swelling. An ultrasonographic study of her hands and feet only disclosed mild peritendinitis without joint synovitis in the fifth PIP joint of the right hand. A diagnosis of arthralgia suspicious for progression to RA (clinically suspect arthralgia [CSA]), as established based on this clinical picture and the presence of anti-CCP antibodies. The probability of progression to clinical RA seems high in this individual. However, some doubts arise regarding the most appropriate management of the symptoms or the disease at this pre-arthritis stage. Some of the relevant questions are as follows:

*Is This Individual Suffering from Inflammatory Arthralgia with a High Risk of Developing RA in the near Future? If So, Is This Risk Imminent? Can We Quantify This Risk?*

Given the current knowledge, it is widely agreed among rheumatologists that this individual has inflammatory arthralgia, and the presence of an ACPA-positive test suggests a high risk of progression to RA, likely in the near future. This case represents a possible pre-clinical RA, although the diagnosis remains retrospective until persistent clinical arthritis develops. Such individuals have been described in the literature as suffering from inflammatory arthralgia, CSA, or even imminent RA [1,2]. In 2017, EULAR published recommendations for defining individuals with arthralgia suspicious for RA progression, emphasizing the type and characteristics of arthralgia (recent onset, symptoms predominating in the early morning, and affecting small joints of the hands) [3]. This definition requires the absence of clinical joint swelling, differentiating it from undifferentiated arthritis, where patients have clinical arthritis but do not meet criteria for RA or other inflammatory arthritis [4]. In this strict definition, the patient suffering from palindromic rheumatism, a form of intermittent arthritis/periarthritis without joint pain and swelling in the intercritical period, could not be classified as inflammatory arthralgia or CSA, although the risk of RA progression in these patients is quite high [5].

The question arises as to whether the risk for RA progression is imminent in these individuals. It is evident that in addition to the clinical phenotype (inflammatory arthralgia), the presence of an ACPA-positive test increases the probability of RA progression and likely decreases the latency period to persistent arthritis. Several studies have addressed this issue to quantify the risk, concluding that between 20% to 50% of individuals progress to RA after 2–3 years of follow-up [1,6,7,8]. The percentages may be higher if the individuals have other risk factors such as first-degree relatives with RA, overweight, tobacco smoking [9], high titers of ACPA, or the combination of other characteristic autoantibodies of RA, such as RF or anti-CarP [10]. However, it is important to note that most individuals do not progress to RA in the short term. Therefore, from a practical point of view, determining the exact risk in an individual patient is challenging, complicating the recommendation of a therapeutic strategy at this stage.

*Why Does This Individual Have Joint Pain without Evidence of Inflammation at the Clinical Exam, Even Using Sensitive Imaging Techniques Such as Ultrasound or MRI?*

As previously discussed, the definition of CSA or inflammatory arthralgia is based on the absence of synovitis at the physical examination. In some cases, the presence of inflammation may be very mild and difficult to recognize, especially given variability among rheumatologists in assessing clinical synovitis [11] This feature is more relevant in these patients, including those with early arthritis where signs of inflammation may be mild or subtle. However, studies confirm that a high percentage of these individuals present with synovitis when examined using imaging techniques such as MRI or ultrasound, which may explain the presence of joint pain or stiffness [12,13]. These techniques have higher sensitivity than clinical examination, though possible false positives may occur. In approximately half of the individuals with CSA, no clear evidence of imaging synovitis is found [12]. In these cases, no satisfactory explanation exists for the presence of persistent joint pain.

Pain is a complex and subjective symptom that may be influenced by psychological or social factors, but in patients with CSA, the pain is inflammatory and located in joints commonly affected by synovial inflammation. Therefore, it is possible that imaging techniques are not sufficiently sensitive to capture some degrees of synovitis that may have a clinical impact. It is possible that pathological studies of synovial tissue have detected relevant changes, perhaps only at the molecular level, in these individuals [14].

It has been suggested that in patients with ACPA-positive inflammatory arthralgia, autoantibodies “per se” may account for the induction of joint pain in the absence of synovial inflammation [15]. In a rat model, ACPA may induce the production of IL-8 by monocytes, a chemokine that may stimulate sensory neurons and induce joint pain [16]. However, this mechanism may explain pain in ACPA positive patients but does not account for inflammatory arthralgia in seronegative individuals.

*What Is the Most Appropriate Management for This Individual at This Stage? What Is the Scientific Evidence for the Benefits of Antirheumatic Drugs in These Individuals? What Is the Drug of Choice?*

There is no satisfactory answer to these questions. In clinical practice, it seems reasonable for patients with CSA with known risk factors to address these modifiable factors, such as avoiding tobacco consumption, reducing body weight, and maintaining good dental hygiene, although no scientific evidence exists about the exact clinical benefit in this population. However, should these individuals be treated with antirheumatic drugs beyond symptomatic treatments like analgesics, NSAIDs, or even low doses of glucocorticoids? Does the clinical picture of our patient, at risk for RA progression, warrant an antirheumatic drug intervention with synthetic or targeted disease-modifying antirheumatic drugs (DMARDs)?

This question lacks a definitive answer. The choice and use of drugs for our patient would likely differ among rheumatologists. Intensive drug treatment might seem more acceptable in the presence of synovitis determined via imaging techniques but may be met with reluctance in the absence of subclinical synovitis. Furthermore, the goal of therapeutic intervention needs clarification: should it focus on significant improvement in joint pain, disability, or achieving remission, or should it aim to delay or even prevent progression to RA?

The quality of life for RA patients has improved dramatically due to early treat-to-target strategies and the use of targeted therapies that efficiently control synovitis [17]. However, there is no cure for these patients once clinical arthritis develops. At the pre-rheumatoid stage, intensive therapeutic intervention may hypothetically reduce the risk of developing persistent clinical arthritis and provide a “cure”, at least for some individuals. This potential window of opportunity is encouraging.

Recent years have seen several randomized clinical trials aimed at this objective in patients with inflammatory arthralgia, yielding heterogeneous results (Table 1) [18,19,20,21,22,23]. One issue with these studies is the variability in inclusion criteria, and all of them based their criteria for arthralgia on physician opinion or non-validated criteria. None of the studies used the EULAR criteria for arthralgia suspicious for progression to RA [3], as these studies were initiated before the publication of the EULAR recommendations. The study designs also varied based on other inclusion criteria, such as the presence of autoantibodies or synovitis detected by imaging techniques. All trials had a placebo control group and a maximum follow-up period of two years. A withdrawal period in the active arm was included to assess if clinical benefits persisted post-intervention. Three trials involved biologic drugs (abatacept in two and rituximab in one), while methotrexate, hydroxychloroquine, and atorvastatin were each tested in one trial. The latter two were canceled before study completion due to inefficacy [19,20]. The Treat Early trial found methotrexate beneficial for joint pain and disability but did not reduce RA progression compared to placebo at the study’s end [21]. The trials with abatacept (ARIAA and APIPPRA) [22,23] observed positive effects in preventing or delaying RA progression even after the intervention period. However, a significant proportion of patients in the intervention arm developed RA, and many in the placebo arm did not progress to RA. A randomized trial in early-onset palindromic rheumatism (abatacept vs. hydroxychloroquine) is ongoing (EudraCT#: 2017-004543-20). Recent EULAR recommendations on clinical trials for pre-RA or undifferentiated arthritis and palindromic rheumatism were published after the design of these clinical trials [24].

Several questions arise from these trials beyond population inclusion criteria, including the duration of drug therapy, the follow-up period, and the non-interventional periods. Is a 6-month course of abatacept sufficient, as in the ARIAA trial? Or is a single cycle of rituximab, as in the PRAIRI trial, adequate? Is intermittent or continuous drug use necessary to maintain the objective? What is the best drug for these at-risk individuals?

If the goal is to prevent disease progression rather than just improve clinical and functional outcomes, cell-modifying agents such as abatacept or rituximab may be more appropriate than methotrexate, the standard drug used for early RA. However, the challenge of stratifying and quantifying risk, coupled with the fact that many patients do not progress to RA, complicates the design of interventional trials. Moreover, participants may be reluctant to engage in trials for drugs that may raise concerns about adherence, tolerability, and serious adverse events, especially when the aim is to prevent a hypothetical but not certain adverse outcome [25].

Future Perspectives and Research Agenda

The current knowledge highlights significant doubts regarding the optimal management of individuals at risk for RA. However, theoretical intervention at this pre-RA stage might influence the progression to persistent and destructive polyarthritis such as RA. The critical question is whether therapeutic intervention during this “window of opportunity” should be uniform across the entire at-risk population or if it should target a specific subgroup that might benefit more clearly from this strategy. Stratification of at-risk individuals is essential to identify those with very high risk of progression and to avoid overtreatment. Efforts to develop and validate risk stratification scores for clinical practice are ongoing but require further validation [26,27].

A deeper understanding of the immunological mechanisms driving the progression from pre-RA to clinical RA is crucial. Such insights could lead to more rational drug and therapeutic strategies. For example, studies focusing on the types and characteristics of the immune response in seropositive patients (e.g., ACPA titers, isotype use, antigenic expansion, glycosylation, presence of other autoantibodies like RF or anti-CarP) or sensitive innovative imaging findings might better identify individuals with a favorable risk–benefit ratio for therapeutic intervention. Personalized or individualized medicine is vital for this population. Additionally, research into the effects of modifying risk factors such as obesity, smoking, exercise, dietary habits, and even the mucosal microbiome is of particular interest.

In conclusion, there is some scientific evidence suggesting that progression toward clinical arthritis may be delayed or prevented in individuals at risk for RA. However, there remains considerable uncertainty about the best strategy by which to achieve this goal. In the coming decade, new interventional clinical trials involving various antirheumatic drugs in well-defined at-risk populations will provide more insights into this intriguing question, potentially bringing us closer to a hypothetical cure for this disease or at least optimizing the management of our patients.

## Figures and Tables

**Table 1:** Summary of randomized clinical trials evaluating interventions in individuals at risk of rheumatoid arthritis progression.

Trial	Sample Size	Intervention	Dosage	Duration of Active Treatment	Inclusion Criteria	Total Duration of Follow-Up	Primary Outcome	Progression to RA (Active vs. Placebo *)
PRAIRI [18]	82	Rituximab	1000 mg, single-dose	29 months	Arthralgia; ACPA-positive and RF-positive; Subclinical synovitis by MRI or CRP > 0.6 mg/L	29 months	Reduction of ACPA Level > 50%	34% vs. 40%
STAPRA [19]	62	Atorvastatin	40 mg/day	36 months	Arthralgia; ACPA-positive and RF-positive or ACPA-positive with high levels (>3)	Not specified	Clinical Arthritis	29% vs. 19%
STOPRA [20]	144	Hydroxychloroquine	200–400 mg/day	12 months	High ACPA-positive; Subclinical synovitis by MRI	Not specified	2010 ACR/EULAR Criteria for RA; Clinical Arthritis with XR Erosion	34% vs. 36%
TREAT EARLIER [21]	236	Methotrexate	25 mg/week	12 months	Arthralgia; Subclinical synovitis on MRI	24 months	2010 ACR/EULAR Criteria for RA	19% vs. 18%
ARIAA [22]	100	Abatacept	125 mg/week	6 months	Arthralgia; ACPA-positive; Subclinical synovitis by MRI	18 months	Improvement in MRI Scores of Synovitis	35% vs. 57%
APIPPRA [23]	213	Abatacept	125 mg/week	12 months	Arthralgia; ACPA-positive and RF-positive or ACPA-positive with high titer (>3)	24 months	2010 ACR/EULAR Criteria for RA or Clinical Arthritis in >3 Joints	25% vs. 37%

* Note: “Active” refers to the intervention group and “Placebo” refers to the control group.
